# Camptocormia in Parkinson's Disease

**DOI:** 10.4061/2010/267640

**Published:** 2010-06-30

**Authors:** Kazuo Abe, Yutaka Uchida, Masaru Notani

**Affiliations:** ^1^Department of Nursing and Rehabilitation, Konan Women's University, Kobe 657-0001, Japan; ^2^Department of Neurology and Rehabilitation Center, Konan Hospital, Kobe 658-0064, Japan; ^3^Clinical Research Center, Osaka Health Science University, 1-9-27 Tenma, Kita-Ku, Osaka 530-0043, Japan; ^4^Garacia Hospital, Rehabilitation Center, Yokosuka 562-8567, Japan

## Abstract

*Objectives*. Abnormalities of posture represent one of the main features of Parkinson's disease (PD). Among them, camptocormia has been considered as rare in PD. We investigated frequency and clinical features of camptocormia in PD patients. *Methods*. 153 PD patients (mean 68.5 ± 10.7 years old, duration 5.9 ± 2.4 years) outpatiently recruited. After neurologic examination, patients were rated on the Unified PD Rating Scale motor scale (UPDRS Part III), minimental state examination (MMSE). Also we evaluated patients with camptocormia by MRI. Of the 153 PD patients, 27 had camptocormia (mean age, 67.9 ± 7.9 years old; disease duration, 6.1 ± 3.9 years). For further evaluation, we recruited age- and sex-matched 27 PD patients without camptocormia (11 men and 16 women; mean age ±  SD, 69.2 ± 10.1 years, duration 6.0 ± 2.7 years) These selected 54 patients completed several self-assessments. Lumbar and thoracic paraspinal muscles were studied by EMG. *Results*. There were no significant differences in age, duration, severity, and drug dose between patients with and without camptocormia. Analysis of NMSS subitems indicated that PD patients tended to show lower scores for sleep/fatigue, attention/memory, and miscellaneous items. *Conclusions*. We found significant differences concerning nonmotor signs and symptoms evaluated by FAB, PDQ-8, FSQ, VAS-F, and NMSS between patients with and without camptocormia. Our findings indicate that camptocormia is a relatively common sign in PD and that patients with camptocormia scores on the PDQ-8 compared with PD patients without camptocormia. This suggests that improvements in camptocormia of PD patients may improve their QOL.

## 1. Introduction

Parkinson's disease (PD) may involve skeletal abnormalities including extreme neck flexion (“dropped head") and truncal flexion (camptocormia) [1]. Camptocormia in PD is defined by marked anteroflexion of the trunk, which abates in the recumbent position, with no or minimal response to levodopa [1–4]. The condition is exacerbated by walking and is relieved by sitting, lying in the supine position or by volitionally extending the trunk when the patient leans against a wall or a table. Although early reports often attributed camptocormia to a conversion disorder, it is now accepted as an axial feature of Parkinson's disease [5–7]. However, previous studies found no differences between the parkinsonian clinical signs of PD patients with and without camptocormia, but Bloch et al. and Tiple et al. reported that there were differences in terms of disease duration and severity [6–12]. Magnetic resonance imaging (MRI) and single-photon emission computed tomography (SPECT) findings of the brain with dopamine transporters do not differ for PD patients with and without camptocormia [7, 8]. Two attempts have apparently been made to explain the mechanisms underlying camptocormia [6]. The first considers camptocormia to be primarily a dystonia due to a disorder of the striatum [8, 9]. The other theory relates camptocormia to peripheral mechanisms specifically due to myopathy of the antigravity muscles associated with trunk extension [13]. This is supported by findings of myopathic changes in paraspinal muscles. However, the validity of prior reports has been limited by small sample sizes and short follow-ups [8–16]. Availability of more information on camptocormia in PD would help to improve patients‘ quality of life (QOL). In this epidemiological and clinical study of a large outpatient population of 153 consecutive PD patients, we investigated the prevalence of camptocormia in PD, the relationship of camptocormia with the clinical, especially nonmotor, features of PD and the presence of possible risk factors for developing camptocormia.

## 2. Methods

Approval by the central ethics committee for the full study was initially obtained via the research ethics committee. We reviewed 153 PD patients (mean age 68.5 ± 10.7 years old, disease duration 5.9 ± 2.4 years) who sequentially consulted our clinic in 2005 and diagnosed 27 patients with camptocormia. The diagnosis of PD was made according to the UK Brain Bank Criteria [17]. Camptocormia was defined as an anterior flexion of the thoracolumbar spine of 45° or more appearing in orthostatism or during gait and disappearing in the recumbent position [15]. We excluded patients with scoliosis. We evaluated patients in the off and on condition. We excluded patients with isolated neck flexion (“head drop syndrome") because these patients might have been diagnosed with other disorders including multiple systemic atrophy (MSA) [8, 17]. We excluded also patients with a combination of camptocormia and head drop. All patients had brain MRIs. MRIs were performed with a 3.0 T system (Achieva; Philips Medical Systems, Eindhoven, The Netherlands) using a standard head coil. To exclude other neurological diseases, sagittal T1-weighted inversion recovery (TE/TR = 2000/20 msec, TI = 900 msec) images with acquisition matrix 256 × 256, field of view (FOV) 24 × 24 cm and axial-oblique, parallel to the intercommissural plane and liquid attenuated inversion recovery (TE/TR = 11000/125 msec, TI = 2000 msec). T2-weighted spin echo (SE) (TE/TR = 4931/80 msec) images were acquired with 4 mm slice thickness, acquisition matrix 512 × 256, and FOV 24 × 18 cm. MRIs of the thoracolumbar spine were also performed to exclude abnormalities in that area. Lumbar and thoracic paraspinal muscles were studied by EMG at the levels T3 to T10, and L1 to S4. Additional spinal MRIs were conducted, but we could not find abnormal findings suggesting focal fatty changes in muscles or increased muscle bulks. Statistical analysis was performed using JMP version 5 package (SAS institute Japan, Tokyo). Unless otherwise specified, all data are expressed as means ± SD. Differences between groups were examined by the *t* test. *P* values <.05 were considered statistically significant.

## 3. Results

**Figure 1:**
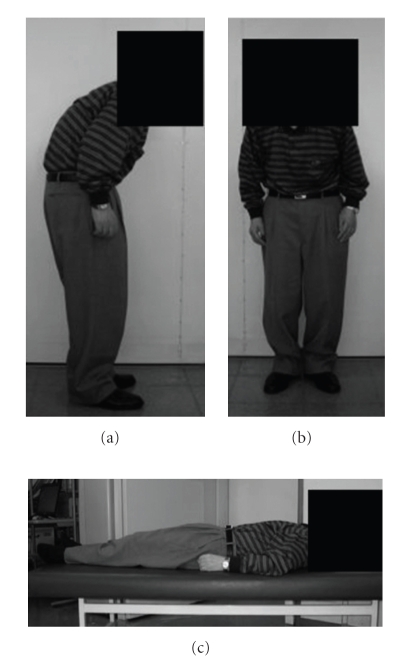
A patient with camptocormia exhibited markedly flexed posture when standing or walking, but was able to lie down in supine position.

Of the 153 PD patients, 27 had camptocormia (mean age, 67.9 ± 7.9 years old; disease duration, 6.1 ± 3.9 years). All PD patients with camptocormia exhibited markedly flexed posture when standing or walking, but were able to sit erect, and were able to extend their trunk fully when facing a wall, straightening their back against it, or lying down in the supine position (Figure 1). Unlike patients with dystonic disorders, these patients have no access to sensory tricks [18]. There are no significant differences between patients with and without camptocormia concerning age, duration, severity, drugs, and drug doses (Table 1). None of the other findings contradicted the diagnosis of PD obtained with MRI. 

For further evaluation, we recruited age- and sex-matched 27 PD patients without camptocormia (11 men and 16 women; mean age ± SD, 69.2 ± 10.1 years)** (** Table 1 **). **After neurologic examination, all patients were rated with the Unified Parkinson's Disease Rating Scale (UPDRS) motor examination (UPDRS Part III) [18], and the minimental state examination (MMSE) [19]. In addition, patients completed the following self-assessments: the frontal assessment battery (FAB) [20], Parkinson's Disease Questionnaires-8 (PDQ-8, a specific instrument for the assessment of health-related quality of life in PD) [21], the hospital anxiety and depression scale (HADS) [22], the fatigue scale questionnaire (FSQ) [23], a fatigue visual analogue scale (VAS-F, 0 (the worst imaginable fatigue state) to 100 (no fatigue at all) [24], PD sleep scale (PDSS) [25], and the nonmotor symptom assessment scale (NMSS) for PD [26, 27].

There were no significant differences in age, duration, severity, and drug dose between patients with and without camptocormia, but there were significant in terms of the total scores for FAB, PDQ-8, FSQ, VAS-F, and NMSS. Analysis of NMSS subitems indicated that PD patients tended to show lower scores for sleep/fatigue, attention/memory, and miscellaneous items (pain, taste, smell, weight loss, and excessive sweating).

## 4. Discussion

The 17% prevalence of camptocormia in our series is higher than that reported in previous studies [6, 14, 28], and is probably related to differences in the clinical features of the study population. Since we excluded patients who might have parkinsonism other than PD, such as MSA, all our patients had the distinctive clinical features of PD. Our series of 27 patients who all met the criteria for camptocormia, that is, marked flexion of the thoracolumbar spine, which is most prominent while standing and walking and relieved in the supine position, illustrates the broad spectrum of musculoskeletal and neurologic etiologies of this disorder.

The pathogenesis of camptocormia in PD patients is not known [29]. Although some investigators insist that flexion of the neck (dropped head) can also be seen in patients with idiopathic PD, this feature seems to be typically present in patients with multiple system atrophy [30, 31]. In addition to PD, other causes of camptocormia include dystonia and extensor truncal myopathy [9, 13]. Sch*ä*bitz. [12] conducted detailed investigations including EMG, neuroimaging studies of the spine, and muscle biopsy of four parkinsonian patients with camptocormia and found that the pathologic findings were consistent with focal myopathy of the paraspinal muscles. Because we did not find any myopathic changes by EMG, we can rule out the occurrence of these disorders.

Djaldetti et al.'s [6] report deals with eight PD patients who developed camptocormia and found no evident correlation between the severity of camptocormia and levodopa treatment. This was reconfirmed by the results of our study. In some patients the camptocormia posture improved, and in others it was unchanged or even aggravated following levodopa administration. Djaldetti raised the possibility that camptocormia might represent either a rare type of dystonia or an extreme form of rigidity. However, we did not find any evidence of “sensory tricks” used by our patients. In addition, we could not find any radiologic features that could differentiate PD patients with from those without camptocormia. The observation that camptocormia can be associated with lenticular lesions [32] suggests that the striatum and pallidum play an important role in the maintenance of axial posture. In general, however, abnormal intensities in the basal ganglia suggest the possible presence of MSA rather than PD. Since we excluded patients with MSA, patients with abnormal intensities in the basal ganglia may also have been excluded [33–35]. MRI scans of the thoracolumbar spine of our patients showed only degenerative changes.

This systematic assessment of camptocormia in a large sample of PD patients has yielded valid assumptions that provide more information on the relationship between camptocormia and the clinical features of PD. We could not find any differences in severity, duration, L-dopa treatment duration, and daily dose or the presence of dementia between patients with and without camptocormia. In addition, age at the clinical onset of PD, the unilateral/bilateral distribution of symptoms at the onset of PD and at the time of the study, and the development of L-dopa-related motor complications did not significantly differ for patients with and without camptocormia. The lack of correlation between the degree of camptocormia and clinical and treatment-related variables could imply that the pathophysiology of camptocormia involves additional, nondopaminergic mechanisms. In this regard, we found significant differences in nonmotor signs and symptoms evaluated by FAB, PDQ-8, FSQ, VAS-F, and NMSS between patients with and without camptocormia. These assessment scales evaluate fatigue, frontal function and quality of life [6]. Djaldetti et al. found that three patients reported worsening of camptocormia with fatigue. For clinical use, fatigue is best defined as difficulty in initiating or sustaining voluntary activities. Disorders of neuromuscular junction transmission and metabolic diseases cause muscle fatigability, which is characterized by failure to sustain the force of muscle contraction (peripheral fatigue). Fatigue is also seen in diseases that affect the central nervous system, which is characterized by failure to sustain attention or to complete mental tasks (central fatigue) [36, 37]. Fatigue in multiple sclerosis (MS) may be associated with frontal cortex and basal ganglia dysfunction which can result from demyelination of the frontal white matter [38]. These relationships between fatigue severity and existence of camptocormia suggest that development of camptocormia may have some relationship with frontal lobe dysfunction in PD. In support of this hypothesis, our results show that PD patients with camptocormia had lower scores for NMSS subitems related to fatigue, attention and memory, which are thought to be frontal lobe functions [26, 27, 39, 40]. P Clinical and neuroimaging findings have suggested that the frontal lobe of PD patients can be considered to be somewhat dysfunctional [8, 41]. This notion seems to support our hypothesis.

Our findings indicate that camptocormia is a relatively common sign in PD and that patients with camptocormia scores on the PDQ-8 compared with PD patients without camptocormia. This suggests that improvements in camptocormia of PD patients may improve their QOL. However, present therapeutic regimens, including drug therapy, surgical therapy and rehabilitation, have limited effect on camptocormia. Since we believe that the pathogenesis of camptocormia in PD patients may involve a variety of factors, further study is needed to identify these factors, which may then lead to an effective therapy for camptocormia in PD patients.

## Figures and Tables

**Table 1:** Demographic and clinical details of Parkinson's disease patients with and without camptocormia, and controls.

	Controls	PD	PD with camptocormia	PD without camptocormia
Number	27	153	27	27
mean age (±sd) (years)	67.4 ± 8.9	68.5 ± 10.7	69.2 ± 10.1	67.9 ± 7.9
sex (m)		70	11	11
mean duration of disease (years)		5.9 ± 2.4	6.0 ± 2.7	6.1 ± 3.9
drugs (levodopa equivalent doses (mg), (mean ± sd))		432 ± 212	440 ± 221	427 ± 203
Hohen-Yahr staging (mean ± sd)		3.0 ± 0.7	3.1 ± 0.5	3.1 ± 1.6
UPDRS (Part III) (mean ± sd)		34.0 ± 16.0	30.4 ± 5.3	32.5 ± 5.1
MMSE (mean)	28.6	27.8	27.6	27.9
FAB (mean ± sd)	16.2 ± 0.8		15.1 ± 1.5	13.7 ± 1.6 ^#,∗^
PDQ-8 (mean ± sd)			16.0 ± 6.1	20.4 ± 4.6 ^#,∗^
HADS-depression (mean ± sd)without depression ≦8	4.6 ± 3.4		8.6 ± 5.4 ^#^	8.3 ± 5.1 ^#^
HADS-anxiety (mean ± sd) without anxiety ≦11	5.9 ± 4.3		8.0 ± 4.8	8.8 ± 6.4
FSQ (mean ± sd)	2.4 ± 0.8		4.1 ± 1.3^ #^	4.8 ± 1.0 ^#,∗^
VAS-F (mean ± sd)	30.8 ± 10.2		48.0 ± 18.5 ^#^	62.1 ± 17.8 ^#,∗^
PDSS (mean ± sd)	120.0 ± 14.8		70.4 ± 26.0 ^#^	69.5 ± 17.3 ^#^
NMSS (mean ± sd)			46.5 ± 21.1	55.7 ± 15.5*

^#^comparing with controls.

*comparing with PD without camptocormia.
